# Exosomes Derived From CircAkap7-Modified Adipose-Derived Mesenchymal Stem Cells Protect Against Cerebral Ischemic Injury

**DOI:** 10.3389/fcell.2020.569977

**Published:** 2020-10-02

**Authors:** Limin Xu, Haifeng Ji, Yufeng Jiang, Liying Cai, Xiaoyin Lai, Feifei Wu, Rongguo Hu, Xuelian Yang, Huan Bao, Mei Jiang

**Affiliations:** ^1^Department of Clinical Laboratory, Shanghai Gongli Hospital, The Second Military Medical University, Shanghai, China; ^2^Department of Neurology, Shanghai Gongli Hospital, The Second Military Medical University, Shanghai, China; ^3^Department of Clinical Medicine, Clinic Medical College of Anhui Medical University, Hefei, China; ^4^Department of Neurology, Shanghai East Hospital, Tongji University School of Medicine, Shanghai, China

**Keywords:** cerebral ischemic injury, exosomes, circular RNA, autophagy, oxidative stress

## Abstract

**Background:**

Cerebral ischemic injury is a complicated pathological process. Adipose-derived stromal cells (ADSCs) have been used as a therapeutic strategy, with their therapeutic effects chiefly attributed to paracrine action rather than *trans-*differentiation. Studies have shown that circAkap7 was found to be downregulated in a mouse model of transient middle cerebral artery occlusion (tMCAO).

**Methods:**

To explore whether exosomes derived from circAkap7-modified ADSCs (exo-circAkap7) have therapeutic effects on cerebral ischemic injury, a mouse model of tMCAO, as well as an *in vitro* model of oxygen and glucose deprivation-reoxygenation (OGD-R) in primary astrocytes, were used.

**Results:**

Results showed that treatment with exo-circAkap7 protected against tMCAO in mice, and *in vitro* experiments confirmed that co-culture with exo-circAkap7 attenuated OGD-R-induced cellular injury by absorbing miR-155-5p, promoting ATG12-mediated autophagy, and inhibiting NRF2-mediated oxidative stress.

**Conclusion:**

We demonstrate here that exo-circAkap7 protected against cerebral ischemic injury by promoting autophagy and ameliorating oxidative stress.

## Introduction

Stroke is one of the leading causes of fatality worldwide. The pathological mechanisms of stroke are complex, and include excitotoxicity, oxidative stress, ion imbalances, inflammation, and apoptosis (Yenari and Han, 2012). Although recent studies on the pathogenesis of ischemic stroke have resulted in improvements in long-term prognoses, current treatments for acute cerebral ischemia are still largely based on the intravenous administration of recombinant tissue plasminogen activator (Prabhakaran et al., 2015). Due to the short therapeutic time window for this approach, as well as medication contraindications, fewer than 5% of stroke patients undergo this treatment, and risk bleeding outcomes (Kanazawa et al., 2017). The clinical research into neuroprotective drugs for stroke has so far been unsuccessful (Chamorro et al., 2016). According to an epidemiological survey, the incidence of stroke in China is increasing each year, and is as high as 120 in 100,000, with more than 2 million individuals suffering from stroke each year (Wu et al., 2013). Stroke therefore represents a substantial disease burden (Prabhakaran et al., 2015).

Stroke can be divided into ischemic or hemorrhagic categories, of which ischemic stroke accounts for about 80% of cases, and hemorrhagic stroke accounting for the remainder (Rennert et al., 2016). In recent years, many studies have reported that MSCs especially adipose-derived MSCs (ADSCs), have a therapeutic effect on cerebral ischemia and other brain injuries, due to their potential to differentiate and multiply (Huang et al., 2017; Lin et al., 2017; Bi et al., 2018; Cheng et al., 2018). However, there are limitations to treatments involving MSCs, because the inflammatory and anoxic microenvironment of the stroked area results in a high rate of apoptosis, and consequently poorer-than-expected therapeutic effects (Yoshimura et al., 2008). Previous research has demonstrated that cells have the ability to contact each other via the secretion of exosomes. Exosomes are normally kept in intraluminal vesicles, but can be released to combine with the cell wall (Simons and Raposo, 2009). Exosomes can interact with target cells in different ways as a form of intercellular communication, such as the entry into target cells via endocytosis, thereby delivering information in RNA or protein format (Valadi et al., 2007; Cantaluppi et al., 2012). Previous researches showed that miR-30d-5p-enhanced ADSC-derived exosomes prevent cerebral injury by inhibiting autophagy-mediated microglial polarization to M1 (Jiang et al., 2018). Besides, treatment of stroke with tailored exosomes enriched with the miR-17-92 cluster increases neural plasticity and functional recovery after stroke (Xin et al., 2017). Hence, modified exosomes are thus able to influence a variety of physiological and pathological pathways in stroke.

Circular RNAs (circRNAs) are a class of non-coding RNAs that form a closed loop structure (Salzman, 2016). CircRNA can be derived from exons, introns, non-coding regions, or intergenic regions (Memczak et al., 2013; Zhang Y. et al., 2013). High-throughput sequencing and computer analyses have revealed the presence of large amounts of circRNA in mice and humans (Jeck et al., 2013; Rybak-Wolf et al., 2015), with more than 65,731 circRNAs detected in human brain tissue alone (Rybak-Wolf et al., 2015). Studies have also revealed conserved circRNA expression and sequence within neural cells. circRNAs are more stable than linear transcripts (Jeck et al., 2013), and are able to adsorb miRNAs in a sponge-like manner, which can act to separate miRNAs from their mRNA targets, thereby inhibiting miRNA function (Hansen et al., 2013; Memczak et al., 2013; Li L.J. et al., 2017). Bai et al. (2018) reported that a novel circRNA, circDLGAP4, had a low level of expression in the ipsilateral hemispheres of mice with tMCAO, and that overexpression of circDLGAP4 was able to ameliorate cerebral ischemic injury by absorbing miR-143. Another high-throughput circRNA microarray study revealed that mm9_circ_010383 (circAkap7) was suppressed in tMCAO compared to sham surgery, however the specific regulatory mechanism of circAkap7 in cerebral ischemia remains unknown (Mehta et al., 2017). circAkap7 is located in chromosome 10 and translated from the spanning junction ORF formed by the covalent connection of exon 1 and exon 4 of the Akap7gene, which codes for a widely expressed scaffolding protein (Johnson et al., 2012). The primary cellular function of Akap7 is spatial regulation of cyclic adenosine monophosphate (cAMP) signaling via cytoskeletal anchoring of PKA22–24. Alternative splicing results in multiple Akap7 isoforms which are functionally diverse within the context of A-kinase signaling (Trotter et al., 1999). Previous study showed that Akap7 is widely expressed throughout the brain (Jones et al., 2016) and Akap7 expression levels may have clinical utility as a prognostic biomarker for post-stroke BBB complications (O’Connell et al., 2017). Our present study found that overexpression of circAkap7 and other circRNAs enhanced cell viability in OGD-R cells (unpublished data). Therefore, exo-circAkap7 was used for further research.

In our study, the levels of circAkap7 were assessed in *in vitro* and *in vivo* models of cerebral ischemia and used to investigate circAkap7 effects and mechanisms of action for the treatment of ischemic injury using ADSC-derived exosomes.

## Results

### Treatment With Exosomes Derived From CircAkap7-Modified Adipose-Derived Mesenchymal Stem Cells Ameliorates Cerebral Ischemic Injury

**FIGURE 1:**
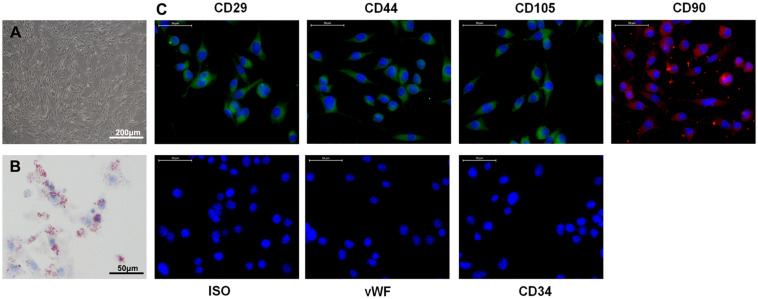
Characteristics of ADSCs. **(A)** Typical fibroblastic-like morphology of ADSCs. **(B)** Adipogenesis was detected on day 18 using Oil Red O staining. **(C)** Assessment of cell surface markers by immunofluorescence staining. ADSCs were positive for MSC markers CD29, CD44, CD90, and CD105, but negative for endothelial markers CD34 and vWF. Negative isotype controls are shown. Nuclei were counterstained with DAPI (blue). Scale bars = 50 mm.

**FIGURE 2:**
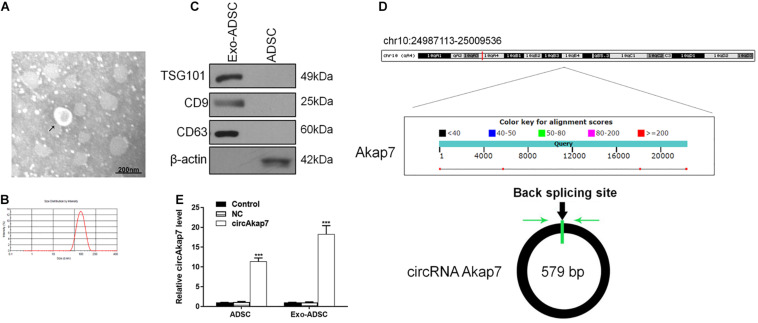
Identification of exosomes. **(A)** TEM image of exosomes. **(B)** Results of nanoparticle tracking analysis of exosomes. **(C)** Western blotting for CD63 and TSG101 as markers of ADSC-derived exosomes. **(D)** The genomic loci of the Akap7 gene and circAkap7. Green arrow indicates back-splicing. **(E)** RT-PCR detection showing expression of circAkap7 in ADSCs and exosomes after transfection. Data are presented as the mean ± SD, each experiment was repeated six times. ***p < 0.001 vs. control group.

Accumulating evidence indicates that exosomes derived from ADSCs have the ability to treat various diseases, including diabetes (Zhao et al., 2018), stress urinary incontinence (Ni et al., 2018), amyotrophic lateral sclerosis (Lee et al., 2016), and ischemia/reperfusion injuries (Pu et al., 2017). To examine their effect on a model of ischemic injury, we isolated primary ADSCs from C57/Bl6 mice. These ADSCs had a typical fibroblastic-like morphology (Figure 1A), and Oil Red O staining confirmed they were undergoing adipogenesis (Figure 1B). Figure 1C shows that the ADSCs were positive for the MSC markers CD29, CD90, CD44, and CD105, and negative for the endothelial markers CD34 and von Willebrand factor (vWF), as assessed by immunostaining. TEM assessment showed that exosomes purified from ADSCs were 30–100 nm in diameter (Figure 2A), and the size distribution was determined to be slightly below 100 nm using dynamic light scattering (Figure 2B). Western blotting confirmed the expression of exosome markers CD63, CD9, and TSG101 (Figure 2C). Previous studies have demonstrated that circAkap7 is downregulated in tMCAO mice compared to sham mice (Mehta et al., 2017). circAkap7 (mmu_circ_0000154, mm9_circ_010383) is derived from exon 2 of the Akap7gene, located on chromosome 10 (1024987113-25009536), whose spliced mature sequence length is 579 base pairs (Figure 2D). We transfected ADSCs cultured in serum-free media with a circAkap7 overexpression vector for 48 h. Reverse transcriptase-polymerase chain reaction (RT-PCR) results showed that the levels of circAkap7 in ADSCs and ADSC-exosomes were increased following transfection with the circAkap7 vector (Figure 2E).

**FIGURE 3:**
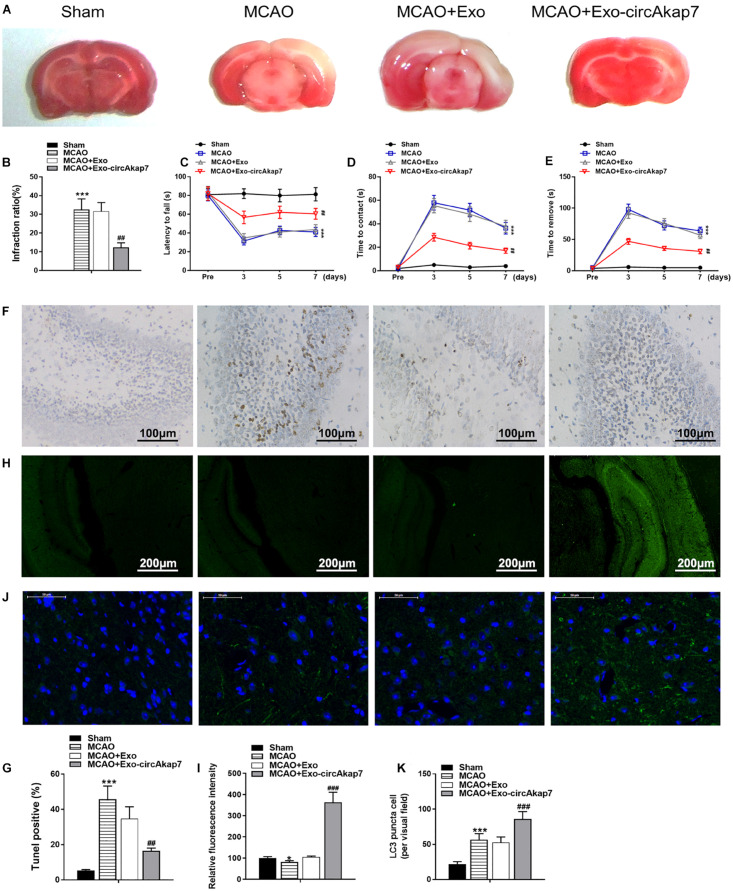
Treatment with ADSC-derived circAkap7-enriched exosomes ameliorates cerebral ischemic injury in a mouse model of tMCAO. **(A,B)** Images showing representative TTC staining. The infarct size was measured and calculated as a percentage of the total area. **(C)** Rotarod test. **(D,E)** Adhesive removal test. Data are expressed as latency to contact **(D)** and tape removal from the impaired forepaw. **(F,G)** The quantified level of apoptosis in cortex around infarct regions was measured by TUNEL assays. The groups were, from left to right: Sham, MCAO, MCAO+Exo, MCAO+Exo-circAkap7. **(H,I)** Quantified fluorescence intensity from FISH assays, conducted to determine the subcellular localization of circAkap7 in in cortex around infarct regions. The groups were, from left to right: Sham, MCAO, MCAO+Exo, MCAO+Exo-circAkap7. **(J,K)** A representative image of LC3 puncta (green) staining in cortex around infarct regions. Nuclei were counterstained with DAPI (blue). The groups were, from left to right: Sham, MCAO, MCAO+Exo, MCAO+Exo-circAkap7. Cells with LC3-positive puncta were quantified, and are expressed as the mean ± SEM, *n* = 8 mice. *p < 0.05, ***p < 0.001 vs. sham, *^##^p <* 0.01, *^###^p <* 0.001 vs. tMCAO.

**FIGURE 4:**
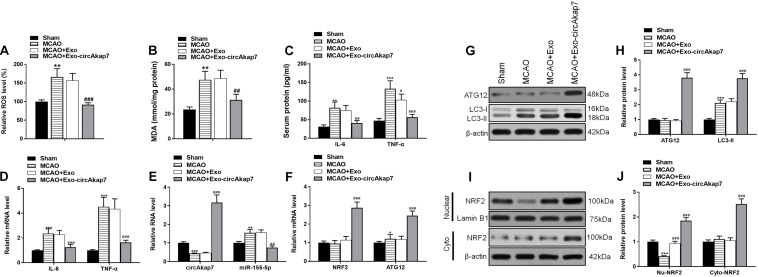
Treatment with exo-circAkap7 attenuates oxidative stress and enhances autophagy. **(A)** Brain ROS levels. **(B)** Brain MDA levels. **(C)** ELISA results showing the serum expression levels of IL-6 and TNF-α in mice. **(D)** RT-PCR results showing the mRNA expression levels of IL-6 and TNF-α in brain tissue. **(E,F)** RT-PCR results showing the levels of circAkap7, miR-155-5p, ATG12, and NRF2 in brain tissue. **(G,H)** The expression of ATG12 and LC3B as analyzed by western blot and quantified. **(I,J)** The expression of cytosolic and nuclear NRF2 as analyzed by western blot and quantified. Data are expressed as the mean ± SEM, *n* = 8 mice. *p < 0.05, **p < 0.01, ***p < 0.001 vs. sham, ^#^*p* < 0.05, ^##^*p* < 0.01, ^###^*p* < 0.001 vs. tMCAO.

We used a tMCAO mouse model to assess the effects of exosomes derived from circAkap7-modified ADSCs (exo-circAkap7) on ischemic injury *in vivo*. Three days after the stroke, TTC results showed that the infarct volume was raised after tMCAO, but exo-circAkap7 treatment reduced the infarct volume (Figures 3A,B). Sensorimotor tests, including the rotarod (Figure 3C) and adhesive removal (Figures 3D,E), consistently revealed impaired neurological performance up to 7 days after the stroke, while exo-circAkap7 treatment improved tMCAO-induced sensorimotor dysfunctions. Results of a terminal deoxynucleotidyl transferase-mediated dUTP-biotin nick end labeling (TUNEL) assay indicated that tMCAO-induced cerebral apoptosis was significant attenuated after injection with exo-circAkap7 (Figures 3F,G). FISH results showed that circAkap7 had infused into the cerebral, and that fluorescence intensity was higher in the exo-circAkap7 group (Figures 3H,I), indicating that circAkap7 could be delivered by exosomes to the cortex. Immunofluorescence staining for the autophagy marker LC3B showed that tMCAO significantly promoted autophagy, and notably, autophagy levels were further increased following exo-circAkap7 administration (Figures 3J,K). Next, we detected the levels of ROS, an indicator of the level of oxidative stress, as well as the levels of malondialdehyde (MDA). Results indicated that the levels of ROS and MDA were significant raised in the tMCAO group, while treatment with exo-circAkap7 reversed this increase (Figures 4A,B). ELISA and RT-PCR results indicated that levels of the inflammatory mediators IL-6 and TNF-α in the serum and cortex of tMCAO mice was decreased after treatment with exo-circAkap7 (Figures 4C,D). Furthermore, we assessed the levels of circAkap7, and its target miRNA, miR-155-5p, which has been reported to play a role in TLE (Huang et al., 2018); as well as the autophagy-related gene ATG12, and oxidative stress-related gene NRF2 in brain tissue. RT-PCR results showed that tMCAO decreased the levels of circAkap7, and increased the levels of miR-155-5p and ATG12, whereas the expression of NRF2 was unchanged. Following treatment with exo-circAkap7, the levels of circAkap7, ATG12, and NRF2 were significantly increased, whereas miR-155-5p was decreased, compared to the tMCAO group (Figures 4E,F). Interestingly, the expression levels of autophagy-related protein LC3-II were the same when assessed by both immunofluorescence and western blotting (Figures 4G,H). Finally, western blotting results showed that in the tMCAO group, the expression of nuclear NRF2 was reduced, whereas the expression of cytosolic NRF2 was increased. The decrease in nuclear NRF2 was reversed after exo-circAkap7 treatment (Figures 4I,J).

### Co-culture With Exo-circAkap7 Attenuates Oxygen and Glucose Deprivation-Reoxygenation-Induced Cellular Injury in Primary Astrocytes

**FIGURE 5:**
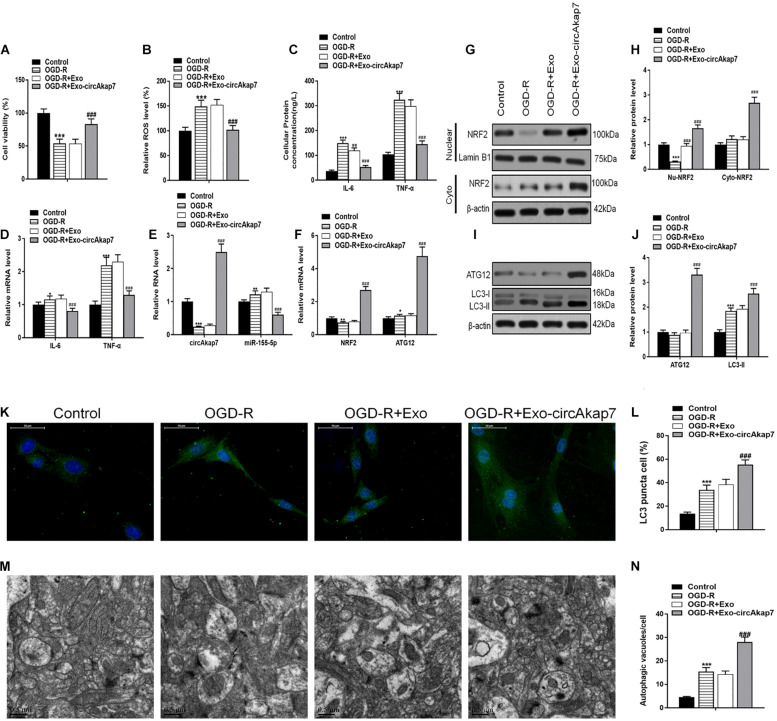
Co-culture with exo-circAkap7 attenuates OGD-R-induced cellular injury in primary astrocytes. **(A)** Cell viability assessed by an MTT assay. **(B)** Detection of cellular ROS levels. **(C)** ELISA results showing the expression of IL-6 and TNF-α in astrocytes. **(D)** RT-PCR results showing the levels of IL-6 and TNF-α in astrocytes. **(E,F)** RT-PCR results showing the levels of circAkap7, miR-155-5p, ATG12, and NRF2 in astrocytes. **(G,H)** The expression of cytosolic and nuclear NRF2 as analyzed by western blot and quantified. **(I,J)** The expression of ATG12 and LC3B as analyzed by western blot and quantified. **(K,L)** Representative photomicrographs and quantification of LC3B-positive puncta in cells. Nuclei were counterstained with DAPI (blue). **(M,N)** Autophagic vacuoles (autophagosomes) as detected by TEM. Representative TEM images are shown, with typical autophagosomes marked with black arrows. The number of autophagosomes per cell was calculated by counting the number of double-membrane organelles in 10 cells. Data are expressed as the mean ± SEM, each experiment was repeated six times. *p < 0.05, **p < 0.01, ***p < 0.001 vs. control, ^##^*p* < 0.01, ^###^*p* < 0.001 vs. OGD-R.

To investigate the therapeutic effects of exo-circAkap7, we isolated primary astrocytes from the cerebral cortex of mice in oxygen and generated a cell glucose deprivation-reoxygenation (OGD-R) mode. Astrocytes were cultured in an OGD condition for 6 h and then co-cultured with exosomes in the 4 h reperfusion phase. An assessment of cell viability showed that OGD-R-induced cell death was ameliorated by administration of exo-circAkap7 (Figure 5A). As shown in Figure 5B, OGD-R increased the ROS levels in primary astrocytes, whereas co-culture with exo-circAkap7 suppressed this increase. ELISA and RT-PCR results indicated that the levels of the inflammatory factors IL-6 and TNF-α in cells were enhanced by OGD-R, and decreased after treatment with exo-circAkap7 (Figures 5C,D). Next, we assessed the levels of circAkap7; its target miRNA, miR-155-5p; ATG12; and NRF2 in astrocytes. RT-PCR results showed that OGD-R decreased the levels of circAkap7 and NRF2, and increased the levels of miR-155-5p and ATG12. After co-culture with exo-circAkap7, the levels of circAkap7, ATG12, and NRF2 were significantly increased, whereas miR-155-5p was decreased compared to the OGD-R group (Figures 5E,F). Furthermore, western blot results showed that in the OGD-R group, the expression of nuclear NRF2 was reduced, and the expression of cytosolic NRF2 was increased, whereas this effect was reversed following exo-circAkap7 treatment (Figures 5G,H). The levels of LC3 in astrocytes were then determined by western blotting. The results again demonstrated that exo-circAkap7 further increased the OGD-R-induced levels of LC3 (Figures 5I,J). Finally, immunofluorescence and TEM analyses indicated that OGD-R treatment markedly enhanced the generation of autophagy plaques in primary astrocytes, and exo-circAkap7 administration increased these levels even further (Figures 5K–N).

### CircAkap7 Functions as a miRNA Sponge, and Is Negatively Regulated by miR-155-5p

**FIGURE 6:**
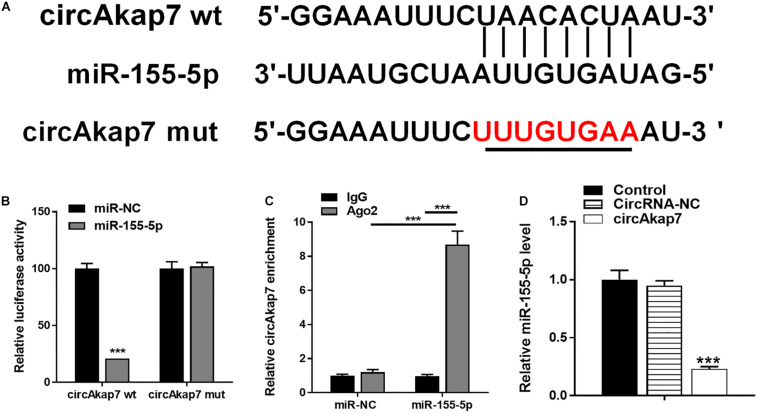
circAkap7 functions as a miRNA sponge to negatively regulate miR-155-5p. **(A)** Wt or mut circAkap7 transfected into astrocytes with or without synthetic miR-155-5p mimics. **(B)** Relative luciferase activity as detected by luciferase assay in astrocytes. **(C)** Anti-Ago2 RIP was performed in astrocytes transfected with miR-155-5p mimics or miR-NC, followed by RT-PCR to detect circAkap7. **(D)** Cellular miR-155-5p expression was determined by RT-PCR. Data are expressed as the mean ± SEM, each experiment repeated three times. ***p < 0.001 vs. miR-NC.

To explore the association between miR-155-5p and circAkap7, we detected the putative miR-155-5p binding sites in circAkap7 (Figure 6A), and made luciferase reporter constructs in which these putative binding sites were mutated. Mutant (mut) and wild-type (wt) luciferase reporter constructs were transfected into astrocytes along with miR-155-5p mimics or negative control miRNAs (miR-NC). Expression of the miR-155-5p suppressed luciferase activity in cells transfected with wt constructs, while luciferase activity was not affected in cells transfected with mut constructs (Figure 6B), indicating that miR-155-5p directly targets circAkap7. Previous studies have demonstrated that miRNAs inhibit translation and reduce mRNA levels in an argonaute-2 protein (Ago2)-dependent manner, by binding to their targets (Yang and Lai, 2011). To examine the role of Ago2 in our system, we performed anti-Ago2 immunoprecipitation (IP) assays in astrocytes overexpressing miR-155-5p, pulling down circAkap7 using an anti-Ago2 antibody, using an anti-IgG antibody as control. We then performed RT-PCR to determine the expression levels of circAkap7. The circAkap7 IP with anti-Ago2 was enriched in astrocytes transfected with the miR-155-5p mimic, compared to those transfected with miR-NC (Figure 6C). Furthermore, the overexpression of circAkap7 significantly reduced the expression of miR-155-5p (Figure 6D).

### Co-culture With Exo-circAkap7 Attenuates Oxygen and Glucose Deprivation-Reoxygenation-Induced Cellular Injury by Inhibiting miR-155-5p

**FIGURE 7:**
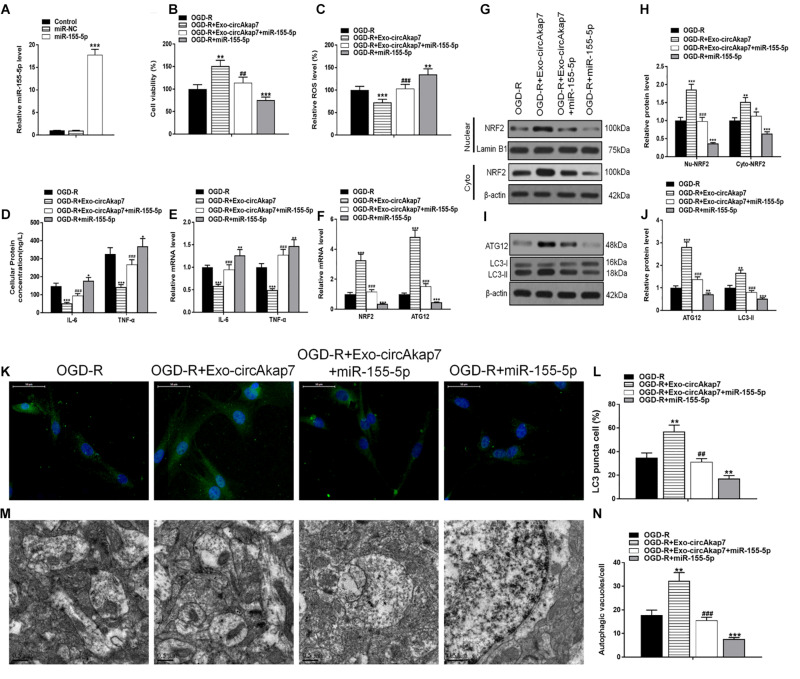
Co-culture with exo-circAkap7 attenuates OGD-R-induced cellular injury by inhibiting miR-155-5p. **(A)** RT-PCR results displaying the level of miR-155-5p in astrocytes. Each experiment was repeated three times. **(B)** Cell viability as assessed by MTT assay. **(C)** Detection of cellular ROS levels. **(D)** ELISA results showing the expression of IL-6 and TNF-α in astrocytes. **(E)** RT-PCR results showing the levels of IL-6 and TNF-α in astrocytes. **(F)** RT-PCR results showing the levels of ATG12 and NRF2 in astrocytes. **(G,H)** The expression of cytosolic and nuclear NRF2 as analyzed by western blot and quantified. **(I,J)** The expression of ATG12 and LC3B as analyzed by western blot and quantified. **(K,L)** Representative photomicrographs and quantification of LC3B-positive puncta in cells. Nuclei were counterstained with DAPI (blue). **(M,N)** Autophagic vacuoles (autophagosomes) as detected by TEM. Representative TEM images, with typical autophagosomes marked with black arrows. The number of autophagosomes per cell was calculated by counting the number of double-membrane organelles in 10 cells. Data are expressed as the mean ± SEM, each experiment was repeated six times. *p < 0.05, **p < 0.01, ***p < 0.001 vs. OGD-R, ^#^*p* < 0.05, ^##^*p* < 0.01, ^###^*p* < 0.001 vs. OGD-R+exo-circAkap7.

The miR-155-5p mimic vector (miR-155-5p) was then transfected into astrocytes for 48 h prior to analysis by RT-PCR (Figure 7A), in order to evaluate the role of miR-155-5p in the process of OGD-R. Cellular viability of astrocytes was measured using an MTT assay. Results showed that the miR-155-5p mimic reversed the therapeutic effects of exo-circAkap7, which worsened the cellular injury induced by OGD-R (Figure 7B). Furthermore, we found that the reduction in ROS levels decreased by exo-circAkap7 was reversed by miR-155-5p, and miR-155-5p increased ROS levels compared to the OGD-R group (Figure 7C). ELISA and RT-PCR results indicated that levels of the inflammatory factors IL-6 and TNF-α in astrocytes were decreased after treatment with exo-circAkap7, while miR-155-5p enhanced this suppression (Figures 7D,E). We then assessed the relationship between miR-155-5p, NRF2, and ATG12. As shown in Figure 7F, the increase in NRF2 and ATG12 induced by exo-circAkap7 was reduced by miR-155-5p. Furthermore, the western blot results showed that the expression of nuclear and cytosolic NRF2 was increased after exo-circAkap7 treatment, while miR-155-5p reduced the exo-circAkap7-induced increase (Figures 7G,H). The levels of ATG12 and LC3 in astrocytes were then determined by western blotting. Results demonstrated that miR-155-5p inhibited the levels of ATG12 and LC3, while exo-circAkap7 enhanced the OGD-R-dependent increase in ATG12 and LC3 (Figures 7I,J). Immunofluorescence and TEM analyses indicated that co-culture with exo-circAkap7 enhanced the generation of autophagy plaques in primary astrocytes, whereas miR-155-5p reduced the generation of plaques (Figures 7K–N).

### The miR-155-5p Directly Targets and Inhibits ATG12 and NRF2

**FIGURE 8:**
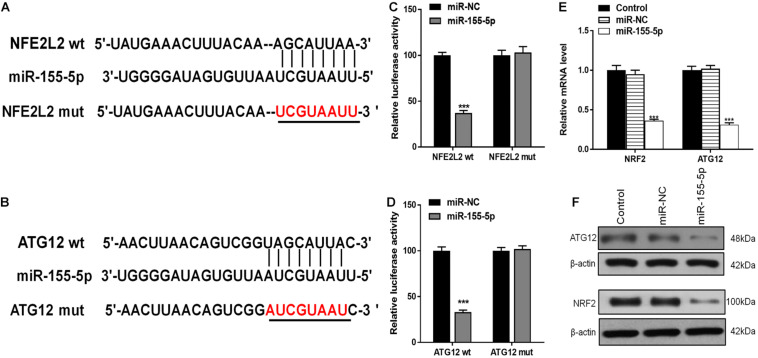
miR-155-5p directly targets and inhibits ATG12 and NRF2. **(A,B)** The predicted binding sites of miR-155-5p in the 3′-UTR of ATG12 and NRF2. The mutated version of the ATG12 and NRF2 3′-UTR is shown. **(C,D)** Relative luciferase activity as detected by luciferase assays in astrocytes. **(E,F)** Cellular ATG12 and NRF2expression as determined by RT-PCR and western blotting. Data are expressed as the mean ± SEM, each experiment was repeated three times. ***p < 0.001 vs. miR-NC.

Using TargetScan, we were able to predict that NRF2 and ATG12 were target genes of miR-155-5p. To confirm this, we generated luciferase reporter constructs containing either the wt 3′-untranslated region (UTR) of NRF2 and ATG12, or a mutated putative miR-155-5p binding site in the 3′-UTR (Figures 8A,B). The constructs were co-transfected into astrocytes along with miR-155-5p mimics or miR-NC, and luciferase activity was detected. Results showed that miR-155-5p overexpression suppressed the activity of the wt group, but not that of the mut or NRF2/ATG12 promoter-deleted groups, indicating that miR-155-5p interacts with the NRF2 and ATG12 3′-UTR to inhibit NRF2 and ATG12 at the post-transcriptional level (Figures 8C,D). RT-PCR and western blot results showed that miR-155-5p inhibited NRF2 and ATG12 at both the mRNA and protein levels, respectively (Figures 8E,F).

### Co-culture With Exo-circAkap7 Attenuates Oxygen and Glucose Deprivation-Reoxygenation-Induced Cellular Injury by Promoting ATG12-Mediated Autophagy

**FIGURE 9:**
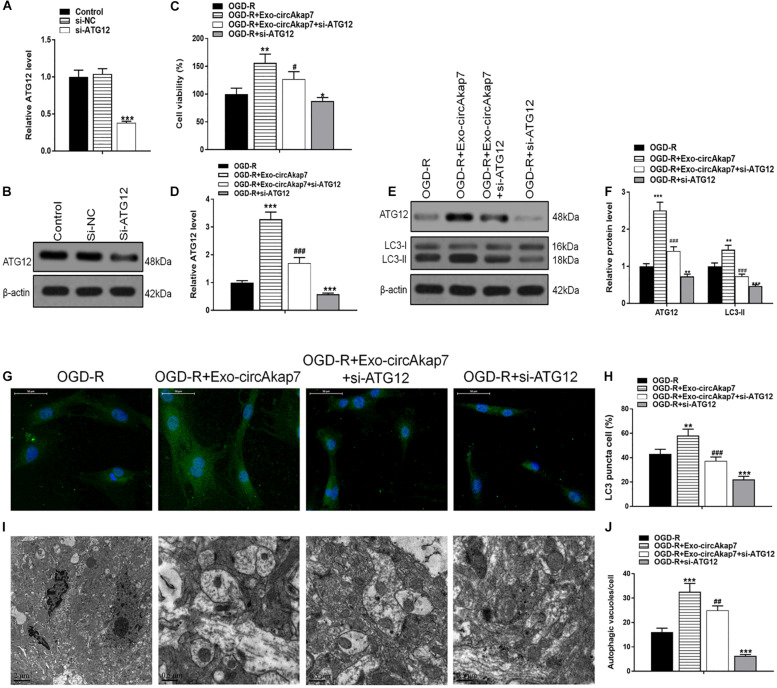
Co-culture with exo-circAkap7 attenuates OGD-R-induced cellular injury by promoting ATG12-mediated autophagy. **(A,B)** RT-PCR and western blot results showing the levels of ATG12 in astrocytes. Each experiment was repeated three times. **(C)** Cell viability as assessed by an MTT assay. **(D)** RT-PCR results showing the level of ATG12 in astrocytes. **(E,F)** The expression of ATG12 and LC3B as analyzed by western blot and quantified. **(G,H)** Representative photomicrographs and quantification of puncta positive for LC3B immunofluorescence in cells. Nuclei were counterstained with DAPI (blue). **(I,J)** Autophagic vacuoles (autophagosomes) as detected by TEM. Representative TEM images are shown, with typical autophagosomes marked with black arrows. The number of autophagosomes per cell was calculated by counting the number of double-membrane organelles in 10 cells. Data are expressed as the mean ± SEM, each experiment was repeated six times. *p < 0.05, **p < 0.01, ***p < 0.001 vs. OGD-R, ^#^*p* < 0.05, ^##^*p* < 0.01, ^###^*p* < 0.001 vs. OGD-R+exo-circAkap7.

To investigate whether exo-circAkap7 acted via ATG12, we generated a siRNA against ATG12: si-ATG12. As expected, both RT- PCR and western blot results showed that si-ATG12 significantly downregulated ATG12 in astrocytes (Figures 9A,B). Cell viability of astrocytes was then measured using an MTT assay. As show in Figure 9C, si-ATG12 reversed the therapeutic effects of exo-circAkap7, which worsened the cellular injury induced by OGD-R (Figure 9C). Next, RT-PCR and western blot results revealed that the increase in ATG12 induced by exo-circAkap7 was reduced by si-ATG12 (Figures 9D–F). The levels of LC3 in astrocytes were then determined by western blotting. Results showed that exo-circAkap7 enhanced the OGD-R-induced levels of LC3, while si-ATG12 reversed this increase (Figure 9E). Immunofluorescence and TEM analyses indicated that co-culture with exo-circAkap7 enhanced the generation of autophagy plaques in primary astrocytes, which were reduced by si-ATG12 (Figures 9G–J).

### Co-culture With Exo-circAkap7 Attenuates Oxygen and Glucose Deprivation-Reoxygenation-Induced Cellular Injury by Promoting NRF2-Mediated Oxidative Stress and Inflammatory Responses

**FIGURE 10:**
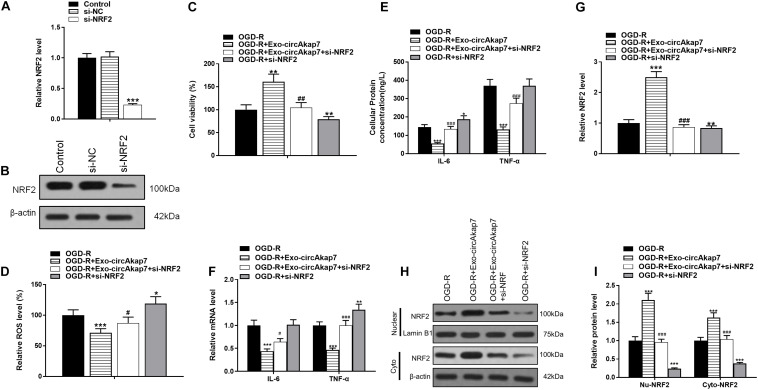
Co-culture with exo-circAkap7 attenuates OGD-R-induced cellular injury by promoting NRF2-mediated oxidative stress and inflammatory responses. **(A,B)** RT-PCR and western blot results showing the levels of NRF2 in astrocytes. Each experiment was repeated three times. **(C)** Cell viability as assessed by an MTT assay. **(D)** Detection of cellular ROS levels. **(E)** ELISA results showing the expression of IL-6 and TNF-α in astrocytes. **(F)** RT-PCR results showing the levels of IL-6 and TNF-α in astrocytes. **(G)** RT-PCR results showing the levels of NRF2 in astrocytes. **(H,I)** The expression of cytosolic and nuclear NRF2 as analyzed by western blotting and quantified. Data are expressed as the mean ± SEM, each experiment was repeated six times. *p < 0.05, **p < 0.01, ***p < 0.001 vs. OGD-R, ^#^*p* < 0.05, ^##^*p* < 0.01, ^###^*p* < 0.001 vs. OGD-R+exo-circAkap7.

To investigate whether exo-circAkap7 acted via NRF2, we constructed an siRNA against NRF2, si-NRF2. As expected, both RT-PCR and western blot results showed that si-NRF2 significantly downregulated NRF2 in astrocytes (Figures 10A,B). Exo-circAkap7 increased the cellular viability of astrocytes, whereas si-NRF2 reversed the exo-circAkap7-induced increase in viability (Figure 10C). Furthermore, we found that ROS levels, which were decreased by exo-circAkap7 administration, were increased by administration of si-NRF2, relative to the OGD-R group (Figure 10D). ELISA and RT-PCR results revealed that the levels of IL-6 and TNF-α in astrocytes were reduced following co-culture with exo-circAkap7, while si-NRF2 reversed this suppression (Figures 10E,F). Finally, we performed RT-PCR and western blotting to determine the mRNA and protein levels of NRF2 in both nuclear and cytosolic cellular fractions. Results revealed that the increase in NRF2 induced by exo-circAkap7 was reduced by si-NRF2 (Figures 10G–I).

## Discussion

Exosomes are vesicles which contain a variety of macromolecular substances, including proteins, mRNAs, miRNAs and circRNAs, and therefore have the ability to influence intracellular signaling pathways (Raposo and Stoorvogel, 2013). Past studies have revealed that ADSC transplantation alone is relatively inefficient in the treatment of ischemic injury (Teng et al., 2015; Wang et al., 2018). The aim of the current study was therefore to attempt to improve the efficiency of treatment of cerebral ischemic injury using ADSC-derived exosomes. To examine the potential protective effects of exosomes on cerebral ischemic injury, we used a mouse model of tMCAO. Our results revealed that treatment with exo-circAkap7 ameliorated cerebral ischemic injury *in vivo*, as well as astrocytic injury *in vitro*, by attenuating oxidative stress and promoting autophagy. Interestingly, several studies demonstrated that treatment of exosome along has the therapeutic effect on stroke (Zhang and Chopp, 2016; Hong et al., 2019). However, our study showed that treatment of stroke with exosomes along had no effect. We think this may be related to the difference in MCAO modeling methods, the times of exosomes injections and the concentration of exosomes.

In recent years, high-throughput sequencing has revealed the presence of large amounts of circular RNA in eukaryotic cells, including in humans (Salzman et al., 2012; Jeck et al., 2013; Memczak et al., 2013; Guo et al., 2014; Zhang et al., 2014). More than 10% of genes in all tested cells or tissues are capable of producing circRNA (Jeck et al., 2013; Guo et al., 2014; Westholm et al., 2014; Fan et al., 2015; Ivanov et al., 2015). Moreover, many circRNAs are highly abundant and have cell or tissue specificity (Liang and Wilusz, 2014; Kelly et al., 2015; Starke et al., 2015). Previous studies have demonstrated that circAkap7 is highly enriched in various brain regions (Rybak-Wolf et al., 2015), and studies have further suggested that circRNA can act as an RNA “sponge” to absorb miRNAs (Hansen et al., 2013; Kelly et al., 2015; Yang W. et al., 2016; Zheng et al., 2016; Chen et al., 2017) and bind proteins (Du et al., 2016; Holdt et al., 2016). In our study, we demonstrated that circAkap7 displayed an endogenous sponge-like effect on miR-155-5p in astrocytes, and that circAkap7 could bind directly to miR-155-5p in an Ago2-dependent manner, indicating that circRNAs could compete with endogenous RNAs.

Several studies have found abnormal expression of miR-155-5p in different diseases, including hepatocellular carcinoma (Lin et al., 2018), renal cell carcinoma (Zhang et al., 2018), atherosclerosis (Chen et al., 2019), vulvar lichen sclerosis (Ren et al., 2018), and TLE (Huang et al., 2018). In our study, we found that the levels of miR-155-5p were upregulated in brain tissue from tMCAO mice compared to sham mice. After treatment with exo-circAkap7, however, miR-155-5p levels were decreased. The use of an miR-155-5p mimic confirmed that miR-155-5p played an important role in the effects of exo-circAkap7. Moreover, bioinformatics prediction and luciferase reporter assays revealed that the ATG12 and NRF2 3′-UTR shared identical miR-155-5p response elements, which were able to bind competitively to miR-155-5p. Our results demonstrated that the miR-155-5p mimic inhibited mRNA and protein expression of ATG12 and NRF2.

Autophagosomes are one of the key components of the intracellular degradation pathway. In the process of autophagy, cells engulf portions of the cytoplasm and organelles, which become autophagosomes upon fusion with lysosomes. Under baseline conditions, cells maintain homeostasis using a variety of measures such as combating metabolic stress, eliminating aging or damaged organelles, and degrading abnormally accumulated proteins. Autophagy is an essential process in the maintenance of cellular homeostasis (Klionsky et al., 2016; Levine and Kroemer, 2019), but it can also be rapidly induced in response to variations in the internal and external state of the cell, such as during starvation, hypoxia, accumulation of metabolites, or damage to organelles. Many neurological diseases involve alterations in autophagy (Levine and Kroemer, 2008; Moloudizargari et al., 2017), and a large body of research indicates that autophagy is invoked during cerebral ischemia-reperfusion injury (Zhang X. et al., 2013; Li et al., 2014; Zhang et al., 2019). Promoting autophagy during brain reperfusion may also contribute to mitophagy-related mitochondrial clearance and the suppression of cellular apoptosis. In the current study, we found elevated levels of autophagy markers in brain tissue from tMCAO mice, and exo-circAkap7 administration led to enhanced levels of the autophagy-related gene ATG12, as a result of its ability to absorb miR-155-5p. However, According to the findings of Zhang X. et al. (2013), autophagy has different effect in permanent or tMCAO mice. Inhibition of autophagy by 3-MA reduced the ischemia-induced infarct in permanent middle cerebral artery occlusion and aggravated the ischemia-induced infarct in tMCAO. This study also showed that inhibition of autophagy reinforced neuronal apoptosis. Interestingly, Wang et al. (2019) found Inhibition of autophagy by 3-MA attuned OGD/R-induced neuron cell death which was contrary to Zhang’s findings. Hence, the mechanism of autophagy in stroke is different in different ischemic/hypoxia time or different cells. So, we will focus on the effect of circAkap7 and autophagy on neurons.

Oxidative stress is considered to be one of the key factors associated with cerebral ischemia-reperfusion injury (Yang Y. et al., 2016; Li X. et al., 2017). As a key regulator of oxidative stress, NRF2 is considered one of the most important regulators of the antioxidant signaling pathway. This oxidase-related gene has multiple neuroprotective functions including protecting against oxidative damage, regulating autophagy, inhibiting apoptosis, reducing cytotoxicity, and maintaining intracellular redox homeostasis (Ding et al., 2015; Buendia et al., 2016; Zhang et al., 2017). Dual luciferase reporter assays revealed that NRF2 is a direct target of miR-155-5p. Furthermore, *in vitro* and *in vivo* experiments demonstrated that exo-circAkap7 attenuated oxidative stress and inflammatory responses by promoting the expression of NRF2 via absorbing miR-155-5p. These results indicated that the therapeutic effects of exo-circAkap7 are associated with NRF2 signaling.

## Conclusion

This study provides evidence that circAkap7 functions as a novel therapeutic circRNA by absorbing miR-155-5p. The *in vitro* and *in vivo* results indicated that exo-circAkap7 protected against ischemic injury by promoting ATG12-mediated autophagy and ameliorated oxidative stress by enhancing NRF2 nuclear transcription. These findings suggested that exo-circAkap7 is a potential treatment strategy for cerebral ischemic injury.

## Materials and Methods

### Animals

C57BL/6 mice (weight 25 ± 2 g) were purchased from the Shanghai Laboratory Animal Center of the Chinese Academy of Sciences (Shanghai, China). Experimental animals were fed a standard laboratory diet and had ad libitum access to water. Mice were housed in a controlled environment with a temperature of 22 ± 1°C, 65 ± 5% humidity, and a 12:12 h light/dark cycle. All animal experiments were conducted in accordance with the Institutional Guidelines for the Care and Use of Laboratory Animals of Pudong New Area Gongli Hospital, Shanghai Second Military Medical University. All surgery was performed under sodium pentobarbital anesthesia, and all efforts were made to minimize suffering.

### ADSC Culture

Adipose tissues obtained from euthanized C57BL/6 mice were rinsed in phosphate-buffered saline (PBS) and cut into 1 × 1 mm pieces. After digestion with collagenase, tissues were centrifuged at 4,000 × *g* for 5 min. The resultant cell pellet was then suspended in Dulbecco’s modified Eagle’s media (DMEM) containing 10% fetal bovine serum (FBS), 1% penicillin-streptomycin, and 2 mM L-glutamine. Cells were then cultured for 48 h in a controlled 38°C atmosphere with 5% CO_2_. Cells were transferred to fresh culture media every 3 days. When cells reached ∼90% confluency, they were passaged and used at passage three. Cells were incubated with conjugated monoclonal antibodies against CD29, CD44, CD90, and CD105 to confirm the identity of ADSCs, while isotype-identical antibodies (PharMingen, San Diego, CA, United States) served as controls. ADSCs were then fixed in 1% paraformaldehyde, and a FACSCalibur flow cytometer (BD Biosciences, San Jose, CA, United States) and FlowJo software (FlowJo, Ashland, OR, United States) were used for quantitative analyses. Logarithmic fluorescence intensities were recorded for 10,000–20,000 cells per sample.

### Isolation and Analysis of Exosomes

The NC and circAkap7 overexpression vectors were provided by GenePharma (Shanghai, China), and were transfected into ADSCs at a final concentration of 20 nmol/L using Lipofectamine 3000 (Invitrogen Life Technologies, Carlsbad, CA, United States). ADSCs were collected for analysis of circAkap7 levels at 48 h post-transfection. ADSCs (circAkap7 overexpression, control, and NC groups) at 80–90% confluency were washed with PBS and cultured in microvascular endothelial cell growth media-2, free of FBS. ADSCs were then supplemented with 1× serum replacement solution (PeproTech, Rocky Hill, NJ, United States) for 24 h. To remove dead cells and debris, ADSCs were centrifuged at 300 × *g* for 10 min, followed by 2000 × *g* for 10 min, after which 5 ml of ExoQuick-TC reagent (System Biosciences, Palo Alto, CA, United States) was mixed with 10 ml of supernatant. After centrifugation at 1500 × *g* for 30 min, the exosome-containing pellet was resuspended in nuclease-free water. TRIzol-LS (Invitrogen) and an Exosomal Protein Extraction Kit (Invitrogen) were used for extracting total RNA and protein, respectively. Exosomes were used immediately for experiments or stored at −180°C. A NanoSight LM10 (Malvern Instruments, Malvern, United Kingdom) nanoparticle tracking system was used to determine the sizes of purified exosomes. Western blotting was used to measure CD9, CD63, and TSG101 protein levels. To assess the protein concentration of exosomes, we used a bicinchoninic acid assay kit (Beyotime, Suzhou, China). TEM was performed on a Libra 120 (Zeiss, Oberkochen, Germany) to analyze vesicle ultrastructure.

### Animal Experimentation

Animals were randomly assigned to four groups: (1) Sham, (2) tMCAO, (3) tMCAO+Exo, and (4) tMCAO+Exo-circAkap7. Each group consisted of eight animals. For tMCAO, reperfusion was allowed after 1 h by removal of the monofilament. Body temperature was maintained at 37°C using a heat lamp (FHC, Bowdoinham) during surgery, and for 2 h after the start of reperfusion. Blood pressure and blood gas analysis instruments were used to monitor arterial blood pressure, arterial pH, arterial pCO_2_, and arterial pO_2._ Exosomes (400 μg of protein) were isolated in PBS and administered by intravenous injection via tail vein at the onset of reperfusion during the tMCAO procedure.

### Infarct Volume Measurement

Measurements of infarct volumes were performed as previously described (Yang et al., 2017). Briefly, infarct volume was determined with 2, 3, 5-triphenyltetrazolium chloride (TTC) 72 h post-MCAO. Mouse brain tissue was sliced into thick sections (1-mm-thick coronal sections) and stained with a 2% solution of TTC for 20 min at 37°C, followed by fixation with 4% paraformaldehyde. TTC-stained sections were imaged and analyzed using Image Pro-Plus 5.1 analysis system (Media Cybernetics, New York, NY, United States). Lesion volumes were calculated using the following formula: [total infarct volume-(volume of intact ipsilateral hemisphere–volume of intact contralateral hemisphere)]/contralateral hemisphere volume × 100%.

### Behavioral Tests

Sensorimotor functional recovery after stroke was measured 3, 5, and 7 days after MCAO. All behavioral tests were performed by an investigator blinded to the experimental conditions. The rotarod (IITC Life Science, New York, NY, United States) test was performed to determine sensorimotor coordination. Briefly, mice were placed on an accelerated rotating rod with an increasing speed from 4 to 120 rpm within 5 min. Mice were tested three times daily with a 5-min intermission. Latency to fall off the rotating rod was recorded. The data are expressed as mean values from three trials. The adhesive removal test was also employed. In brief, a rat was placed in a cage for 1 min and adhesive tape (50 mm^2^) was applied to the distal radial region of the right forelimb as a tactile stimulus. The time to contact and time to tape removal were both recorded. Each animal was tested three times with a cutoff time of 120 s per trial. The data are presented as the mean time to contact and mean time to tape removal on each testing day.

### Brain Tissue Collection and Biochemical Analysis

Anesthetized mice were euthanized by cardiac puncture, and allowed to bleed out. Brains were harvested immediately after cardiac puncture. A portion of fresh liver tissue was fixed in 10% buffered formalin, and the remaining tissue was snap frozen in liquid nitrogen and stored at −80°C. Blood samples were kept at room temperature for 2 h. Serum was collected after centrifugation at 840 × *g* for 15 min. Brain MDA and ROS were analyzed using commercial kits, according to manufacturers’ protocols.

### Fluorescence *in situ* Hybridization

Specific probes against the circAkap7 sequence labeled with cy5 were used for FISH, as previously described (Zeng et al., 2018). Nuclei were counterstained with 4,6-diamidino-2-phenylindole (DAPI). All procedures were performed according to the manufacturers’ protocols (Genepharma, Shanghai, China).

### TUNEL Staining

For the quantification of apoptosis, brain tissues were analyzed *in situ* by TUNEL assay, performed with an Apoptosis Detection Kit (POD, Roche, Switzerland), according to the manufacturer’s instructions. Slices of the xenografts (3 μm thick) were deparaffinized and rehydrated with xylene and ethanol, followed by permeabilization with 20 μg/ml proteinase K (Gibco), and inactivation of endogenous peroxidase with 3% H_2_O_2_. The sections were washed with PBS, then immersed in TdT buffer for 60 min at 37°C. Sections were then incubated with anti-digoxigenin peroxidase conjugate for 30 min, followed by peroxidase substrate. Lastly, slices were counterstained with 0.5% (weight/volume) methyl green.

### Immunofluorescence Analysis

Coronal sections (25 μm) were prepared at the level of the dorsal hippocampus (2.50–3.50 mm posterior to bregma). Frozen sections were dried, washed, permeabilized, blocked in 5% goat serum, and incubated overnight with antibodies against LC3B (ab48394, 1:100; Abcam, Cambridge, MA, United States). Immunolabeled sections were washed and incubated with goat secondary antibodies conjugated with Alexa Fluor 594 or Alexa Fluor 488 (Merck Biosciences, Nottingham, United Kingdom). Sections were mounted in media containing DAPI (Vector Laboratories, Burlingame, CA, United States), and images were captured using an inverted fluorescence microscope (Olympus, Tokyo, Japan).

### Enzyme-Linked Immunosorbent Assay

The levels of IL-6 and TNF-α in blood and primary astrocyte culture supernatants were determined using an ELISA kit (Nanjing Jiancheng Bioengineering Institute, Nanjing, China) according to standard protocols.

### Primary Astrocyte Culture

Primary astrocytes were isolated from the cerebral cortex of mice, as previously described (Vermes et al., 1995). Briefly, the dissociated cortical cells were suspended in DMEM-F12 with 100 units/ml penicillin, 100 μg/mL streptomycin, and 15% FBS. Cells were then seeded onto poly-L-lysine-coated culture flasks at a density of 6 × 10^6^ cells/cm^2^. Astrocytes were acquired using the shaking method after 12–14 days of culture. Microglia and oligodendrocytes were removed from the flasks, and astrocytes were dislodged using 0.25% trypsin, and plated in different culture dishes for further analysis.

### Oxygen and Glucose Deprivation Model

Primary astrocytes were subjected to OGD followed by reoxygenation (OGD-R), to mimic an ischemic-like condition *in vitro*. For the OGD condition, culture media were replaced with glucose-free DMEM preincubated in 95% N_2_/5% CO_2_, and cells were maintained in a hypoxic chamber (95% N_2_/5% CO_2_, 37°C) for 6 h. Reoxygenation was achieved by placing OGD-treated cells into glucose-containing DMEM/F12 under normoxic condition for 4 h (OGD-R). Further *in vitro* analysis was performed with OGD-R-treated cell samples. Control astrocytes were maintained in complete DMEM and incubated in normoxic conditions throughout the duration of the experiment (blank control).

### Cell Transfection

For circAkap7 overexpression, circAkap7 overexpression or NC vectors were purchased from GenePharma. Primary astrocytes were transfected with either circAkap7 or NC vectors at a final concentration of 50 nM using Lipofectamine 2000 (Invitrogen), following the manufacturer’s protocol. Cells were then harvested 48 h post-transfection for additional experiments, including assessment of circAkap7 expression.

To assess miR-155-5p expression, an miR-155-5p overexpression vector (miR-mimic) and negative control (miR-NC) were purchased from GenePharma. Primary astrocytes were then transfected with either the miR-155-5p overexpression construct or miR-NC at a concentration of 50 nM using Lipofectamine 2000 (Invitrogen). Primary astrocytes were used for miR-155-5p expression analysis, or other experiments, at 48 h post-transfection.

To assess ATG12 and NRF2 expression, ATG12 and NRF2 siRNA knockdown constructs, and corresponding negative control constructs (siNC), were obtained from GenePharma. L-02 cells were transfected with the siATG12 or siNRF2 vector at a final concentration of 50 nM, using Lipofectamine 2000 (Invitrogen), according to the manufacturer’s protocol.

### Cell Viability Assay

Primary astrocytes were plated into 96-well plates at an initial density of 5,000 cells/well. After attachment, cells were treated with exosomes for 15 min, followed by treatment with the OGD-R procedure. After treatment, cells were incubated with 500 μg/mL MTT for 4 h. Blue formazan was dissolved in 10% SDS/5% isobutanol/0.01M HCl, and plates were scanned on a microplate reader (Thermo Scientific) at 570 nm with 630 nm as a reference. Cell viability was normalized as a percentage, compared to control wells.

### RT-PCR Analysis

After treatment with or without OGD-R, RNA was isolated from primary astrocytes cells using TRIzol reagent (Invitrogen). cDNA was synthesized from 1 μg of total RNA in a reaction volume of 21 μL, using oligo dT18 primers and SuperScript reverse transcriptase. PCR amplification was carried out with Taq DNA polymerase (TaKaRa, Tokyo, Japan) using 1 μL of the first-strand cDNA as template. The amplification reactions were run with 30 thermocycles of 30 s at 94°C, 30 s at 55°C, and 30 s at 72°C. The expression levels were calculated using the 2^ΔΔCT^ method 33.

### Protein Isolation and Western Blot Analysis

Protein (50 μg) from lysed cells was separated by 10% SDS-PAGE, and transferred to nitrocellulose membranes, followed by blocking for 2 h. Next, membranes were incubated overnight with primary antibodies, followed by horseradish peroxidase (HRP)-conjugated secondary antibodies. The protein bands were visualized using ECL Plus Detection Reagent (Applygen, Beijing, China).

### Electron Microscopy

Cells were fixed with 2.5% glutaraldehyde in phosphate buffer and stored at 4°C until embedding. Cells were then post-fixed with 1% osmium tetroxide followed by increasing dehydration gradients of ethanol and acetone. Cells were then embedded in Araldite, and ultrathin sections were obtained (50–60 nm). Sections were collected onto uncoated copper grids, and stained with 3% lead citrate-uranyl acetate. Images were examined with a CM-120 electron microscope (Philips).

### Statistical Analysis

Data were expressed as mean ± standard error of the mean. The significance of differences between groups was evaluated by one-way ANOVA with LSD *post hoc* test, where *p* < 0.05 was considered to indicate statistically significant differences.

## Data Availability Statement

The raw data supporting the conclusions of this article will be made available by the authors, without undue reservation.

## Ethics Statement

The animal study has been examined and certified by the Ethics Committee of Shanghai Gongli Hospital, The Second Military Medical University.

## Author Contributions

LX, HJ, and YJ performed the research and analyzed the results. LX, HJ, and RH discussed the results. LC, XL, and FW edited the manuscript. XY, HB, and MJ designed the research. LX wrote the manuscript. HB supervised the study. All authors read and approved the final manuscript.

## Conflict of Interest

The authors declare that the research was conducted in the absence of any commercial or financial relationships that could be construed as a potential conflict of interest.

## Abbreviations

ADSCs: adipose-derived mesenchymal stem cells
Ago2: argonaute-2
circRNA: circular RNA
FISH: fluorescence *in situ* hybridization
MDA: malondialdehyde
OGD-R: oxygen and glucose deprivation-reoxygenation
ROS: reactive oxygen species
siRNA: small interfering RNA
TEM: transmission electron microscopy
TLE: temporal lobe epilepsy
tMCAO: transient middle cerebral artery occlusion
TTC: 2,3,5-triphenyl-tetrazolium-chloride
TUNEL: terminal deoxynucleotidyl transferase-mediated dUTP-biotin nick end labeling
vWF: von Willebrand factor.
